# Diorcinol D Exerts Fungicidal Action against *Candida albicans* through Cytoplasm Membrane Destruction and ROS Accumulation

**DOI:** 10.1371/journal.pone.0128693

**Published:** 2015-06-05

**Authors:** Ying Li, Wenqiang Chang, Ming Zhang, Xiaobin Li, Yang Jiao, Hongxiang Lou

**Affiliations:** Department of Natural Product Chemistry, Key Lab of Chemical Biology of Ministry of Education, Shandong University, No. 44 West Wenhua Road, Jinan City, Shandong Province, China; Louisiana State University, UNITED STATES

## Abstract

*Candida albicans*, which is the most common human fungal pathogen, causes high mortality among immunocompromised patients. Antifungal drug resistance becomes a major challenge for the management of *Candida* infection. Diorcinol D (DD), a diphenyl ether derivative isolated from an endolichenic fungus, exerted fungicidal action against *Candida* species. In this study, we investigated the possible mechanism of its antifungal activity. The change of membrane dynamics and permeability suggested that the cell membrane was disrupted by the treatment of DD. This was further supported by the evidences of intracellular glycerol accumulation, alteration of cell ultrastructure, and down-regulation of genes involved in cell membrane synthesis. In addition, the treatment of *C*. *albicans* with DD resulted in the elevation of reactive oxygen species (ROS), which caused the dysfunction of mitochondria. These altogether suggested that DD exerted its antifungal activity through cytoplasmic membrane destruction and ROS accumulation. This finding is helpful to uncover the underlying mechanisms for the diphenyl ether derivatives and provides a potential application in fighting clinical fungal infections.

## Introduction

*Candida* species are the fourth most common pathogenic fungus causing nosocomial bloodstream infections in the United States [1]. *Candida*. *albicans* is the most frequently isolated *Candida* species, accounting for up to 63% of the infections [2,3]. Clinical used antifungal drugs often cause side effects, such as nephrotoxicity, hepatotoxicity, haemolytic anaemia, and life threatening arrhythmias due to lack of target specificity [4,5]. In addition, the formation of biofilms dramatically increases the resistance to antimicrobial drugs up to 10–1000 times [6]. The emergence of drug resistance leads to more nosocomial infections and costs [7], which highlights the need of novel and effective antifungal therapeutics.

**Fig 1:**
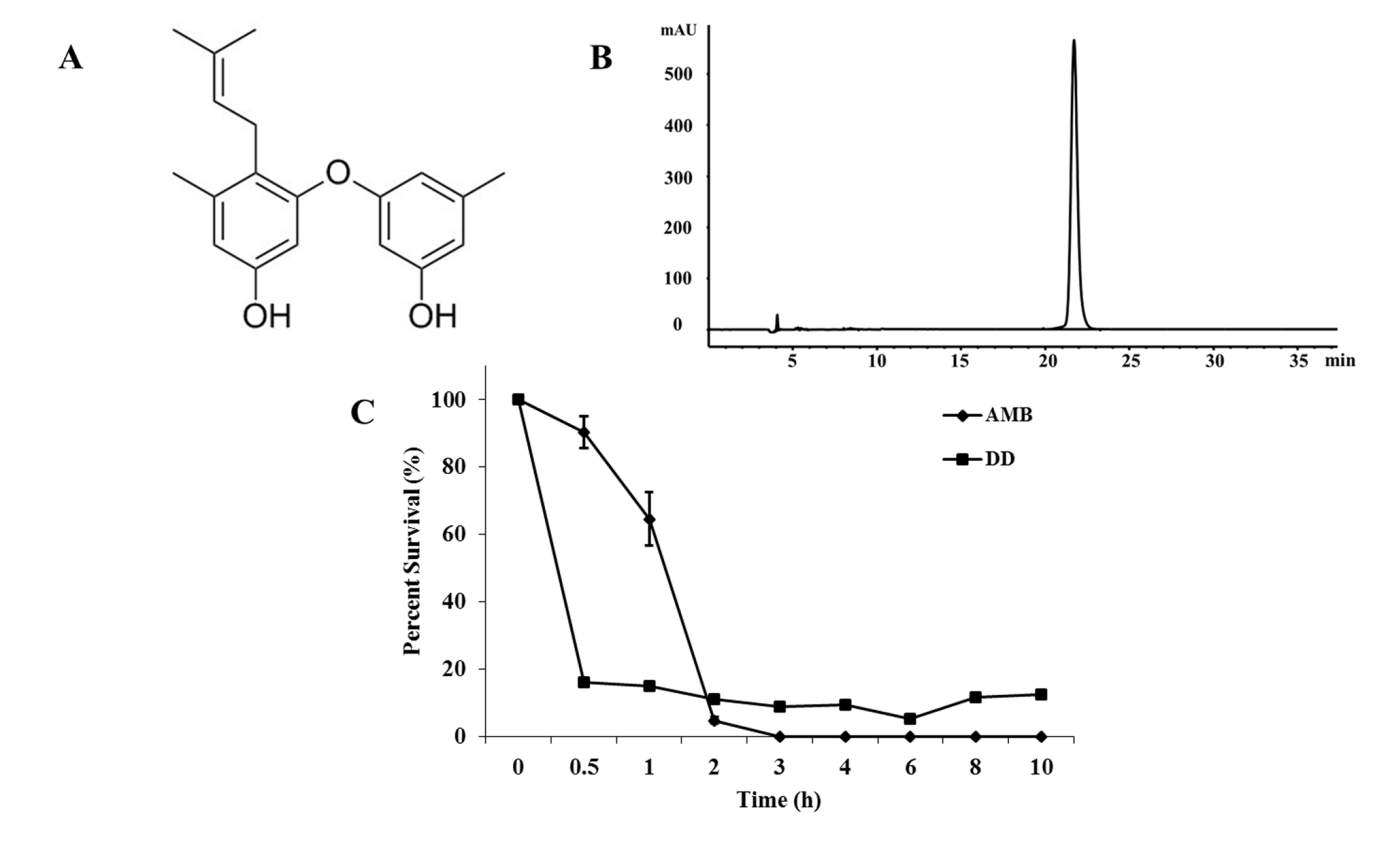
The bioactivity of DD. (A) Chemical structure of DD. (B) The purity of DD as determined by Agilent 1260 system equipped with a ZORBAX SB-C_18_ column in 76% MeOH-H_2_O solvent with a flow of 1.8 mL/min. (C) Time-killing kinetics of DD and AMB at the concentrations of 8 mg/L against *C*. *albicans* SC5314. Bars indicate standard deviations.

Natural products have greatly influenced the development of new therapeutic agents during the past 26 years (1981–2006) [8]. Lichens are symbiotic organisms of fungi, algae, and/or cyanobacteria, and can survive in various environmental conditions [9,10], which provides a possibility of generating secondary metabolites with diversified structures and multiple bioactivities. Fungal endophytes, which reside in internal tissues of living plants without causing any immediate overt negative effects, are an alternative prolific source of bioactive natural products with antimicrobial, antiparasitic, cytotoxic, and neuroprotective activities [11,12]. We focused on extracting biologically active natural products from endolichenic fungi and investigated the underlying mechanism of potential antifungal agents. Diorcinol D (DD) (Fig 1A), a diphenyl ether derivative in this study, was isolated from endolichenic fungus *Aspergillus versicolor* and exerted fungicidal activity against *Candida* species. The existence of fungicidal compounds in endolichenic fungus probably enhanced the viability of their host lichens against pathogenic invasions.

In this study, we aimed to evaluate the activity of DD against *C*. *albicans*, *in vitro*, and clarify its mode of action. Mechanism investigation showed that DD caused the destruction of the cell membrane and elevated intracellular ROS production.

This is the first time that the fungicidal mechanism of a diphenyl ether derivative against *C*. *albicans* has been explored. It will broaden the resources of potential antifungal agents and lay foundations for developing new antifungal drugs.

## Materials and Methods

### Chemicals

DD was separated, in our lab, from lichen endophytic fungus *Aspergillus versicolor*. Amphotericin B (AMB), propidium iodide (PI), fluorescein diacetate (FDA), 1,6-diphenyl-1,3,5-hexatriene (DPH), rhodamine 123 (Rh123), 2′,7′-Dichlorofluorescin diacetate (DCFH-DA), thiourea (Tu), 3-(4,5-dimethyl-2-thiazolyl)-2,5-diphenyl-2-H-tetrazolium bromide (MTT), and 2,3-*bis*(2-methoxy-4-nitro-5-sulfophenyl)2H-tetrazolium-5-carboxanilide (XTT) were all purchased from Sigma (St Louis, MO, USA).

### Strains and culture conditions

*C*. *albicans* wild type strain SC5314 and auxotrophic mutant strain CAI4, clinical isolates of *C*. *krusei* (CK3), *C*. *tropicalis* (CT2), *C*. *glabrata* (CG1), and *C*. *parapsilosis* (CP1) were used in this study. These clinical isolates were kindly provided by the Shandong Provincial Qianfoshan Hospital, Jinan, China. These isolates were stored in physiological saline supplemented with 20% glycerol at -80°C, and subcultured twice on YPD agar plates (2% tryptone, 1% yeast extract, 2% glucose and 2% agar) for 24 h at 30°C. Before each experiment, the cells were prepared in YPD broth (2% tryptone, 1% yeast extract, 2% glucose) for 12 h at 30°C, 200 rpm.

A549 (human lung adenocarcinoma cell line), A2780 (human ovarian carcinoma cell line), MDA-MB-231 (human breast cancer cell line), and HUVEC (human umbilical vein endothelial cell line) were utilized to assay the effect DD on the cell proliferation. These cells were cultured in RPMI-1640 Medium (Hyclone) containing 10% fetal bovine serum supplemented with 100 units/mL of penicillin and 100 mg/L of streptomycin in a humidified atmosphere of 5% CO_2_ at 37°C [13].

### Cell proliferation assay

The effect of DD on A549 (human lung adenocarcinoma cell line), A2780 (human ovarian carcinoma cell line), MDA-MB-231 (human breast cancer cell line), and HUVEC (human umbilical vein endothelial cell line) proliferation was determined by a MTT assay, as previously described [14]. Cells were seeded into 96-well plates and exposed to different concentrations of DD or AMB. After treatment for 24 h, the cells were incubated with MTT for another 4 h in the dark. The spectrophotometric absorbance of each well was measured at 570 nm by a plate reader (Bio-Rad, USA). The IC_50_ values were calculated on the basis of percentage of viable cells. All treatments were repeated at least three times and the results were represented as the mean values ± standard deviations (SDs).

### Time-killing kinetics

To explore the fungicidal action of DD against *C*. *albicans*, the time-killing curves were plotted by measuring the viable cells under the treatment of DD or AMB (positive control). Exponential phased SC5314 cells were diluted with synthetic medium, plus dextrose (SD medium), to 1 × 10^6^ cells/mL. After the incubation with DD or AMB at a final concentration of 8 mg/L, respectively, at 30°C, the number of viable cells was determined by a colony counting method at specific times. The results were represented as the mean values of triplicate measurements from three independent experiments.

### Detection of plasma membrane dynamics

Since DPH could intercalate into lipid bilayers without causing membrane perturbation, we used it to investigate the changes in cytoplasm membrane dynamics under DD treatment. SC5314 cells with an initial density of 1 × 10^6^ cells/mL in SD medium were treated with 0, 4, 8, and 12 mg/L of DD at 30°C for 3 h. The positive control sample was incubated with 8 mg/L of AMB at the same condition. Then, cells were collected and fixed for DPH staining as previously described [15]. The stained cells were measured by a spectrofluorophotometer (Berthold Biotechnologies, Bad Wildbad, Germany) at 350 nm excitation and 425 nm emission wavelengths. All samples were taken in triplicate, and the experiment was repeated three times.

### Detection of membrane permeabilization

To detect the change of membrane permeabilization under DD treatment, cell membrane impermeable fluorescent dye PI was used [15]. SC5314 was exposed to the concentrations of 0 (control), 4, 8, and 12 mg/L of DD in SD medium with 1 × 10^6^ cells/mL. The treatment of 8 mg/L of AMB severed as positive control. After the incubation for 3 h at 30°C, the cells were stained with 5 mg/L of PI for 30 min in the dark. Then, flow cytometry (FACS Calibur; BD Biosciences, San Jose, CA) was used to measure the fluorescence intensity. The fluorescence intensity of cells without PI staining was set to less than 10^1^, and cells with more than 10^1^ fluorescence intensity were regarded as stained ones. The experiment was performed three times, and the results were shown as mean values ± SDs.

### Analysis of cell morphology

The change of morphology was also detected by flow cytometry. SC5314 cells were resuspened at 1 × 10^6^ cells/mL in SD medium and treated with different concentrations of DD at 30°C for 3 h. Changes in cellular morphology were detected by flow cytometry using a forward scatter versus side scatter dot plot to indicate changes in cell size and granularity, respectively.

### Detecting changes in intracellular osmotic pressure

Changes in intracellular osmotic pressure were determined by measuring glycerol content using the Glycerol Assay Kit (Jiancheng, Nanjing, China), according to the provided protocol. Protein concentrations were determined using the Bradford reagent (Beyotime, Shanghai, China) with BSA employed as a standard. The concentrations of glycerol were calculated as the values of the content of glycerol divided by the content of protein. Results are the mean of triplicate experiments ± SDs.

### Transmission electron microscopy (TEM)

To visualize the effect of DD on the *C*. *albicans*’ cell ultrastructure, TEM observation was performed [16]. SC5314 cells were treated by 12 mg/L of DD in SD medium at 30°C for 3 h. Cells without drug treatment served as the control. Cells were harvested by centrifugation at 1000 × g for 5 min. The pellets were fixed, desiccated, and embedded, as previously described [16]. Then, cells were observed under a Hitachi H-800 TEM (Tokyo, Japan).

### Measurement of gene expression

We performed qPCR to quantify the expression of genes, such as high-osmolarity glycerol (HOG) pathway genes (*HOG1* and *RHR2*) and genes involved in biosynthesis of cell membrane and cell wall assembly. Overnight cultured SC5314 cells were diluted to a cell concentration of 5 × 10^6^ cells/mL in SD medium. After the incubation with DD at a final concentration of 8 mg/L for 3 h, the cells were collected and washed by centrifugation at 1500 × g for 3 min at 4°C, and the total RNA was isolated by the hot phenol method, as previously described [17]. cDNA was synthesized using the RT kit (Toyobo Co, Osaka, Japan) according to the manufacturer's instruction. The qPCR was performed using a SYBR green master mix in an Eppendorf Mastercycler Real Time PCR System. The primer sequences are listed in Table 1. The housekeeping gene *18S rRNA* served as the internal reference gene and the data were calculated based on the formula 2^-ΔΔCT^. All samples were run in triplicate.

**Table 1:** Gene-specific primers used for relative quantification of genes expressions by RT-PCR.

Primers	Sequence
*HOG1*-F	GTCTGTGGGTTGTATCTTAG
*HOG1*-R	TCACTAAATGGGATAGGGTC
*RHR2*-F	GCCGTACATTTGATGTCATT
*RHR2*-R	AAAGTACCAGAAGTGACAAC
*ERG1*-F	TGGATAGTGATTCCACATTG
*ERG1*-R	TGTTAGGATCCAGAGGATCA
*ERG3*-F	TTTCATTGTGGCTTACTTATC
*ERG3*-R	AGGAAGGAATACCCATTTAAT
*ERG6*-F	ACAAGCTACTGCTAGACAT
*ERG6*-R	ATCTTGTGATTTCTCTACCAG
*ERG9*-F	TAGAAAGTAGAACATTACCAG
*ERG9*-R	CATACTTGGAGGTAAAGC
*ERG11*-F	TGTCCAAATTC CAGATTAATG
*ERG11*-R	AATAAAGATCTTGAAGCAGTG
*ERG24*-F	ATTACTTGTTACCTGGCAAG
*ERG24*-R	TAATATTCAAGAGAGCTGTCG
*ERG25*-F	AGTGATAAAGAACAATGGGAATGT
*ERG25*-R	TACTGCCCATTGAATCAACATA
*ERG26*-F	TGTAATTGTTCATTCAGCTTC
*ERG26*-R	CATTAAATATCACACCAGCTG
*FKS2*-F	GATCACGAGTCTGTGATTG
*FKS2*-R	AATACATGAGACCAGCCTC
18S rRNA-F	AATTACCCAATCCCGACAC
18S rRNA-R	TGCAACAACTTTAATATACGC

### Measurement of ROS generation

Since, fluorescence probe DCFH-DA could cross cell membranes and be hydrolyzed to nonfluorescent DCFH, which could be rapidly oxidized to highly fluorescent 2’,7’-dichlorofluorescein (DCF) by the intracellular ROS, we used DCFH-DA as an indicator to detect the production of ROS [18]. To investigate the effect of ROS accumulation on the DD mediated antifungal activity, antioxidant Tu that can neutralize ROS generation, and mitochondria inhibitor NaN_3_ that can slow mitochondrial ROS production, were utilized. SC5314 cells were diluted to 1 × 10^6^ cells/mL in SD medium and exposed to different concentrations of DD at 30°C for 3 h. The treatment of 8 mg/L of AMB severed as positive control. Following staining with 40 mg/L of DCFH-DA for 30 min in the dark, the cells were collected, and the fluorescence intensity was measured using flow cytometry, as described previously [19]. The data were processed by WinMDI 2.9 software. In addition, the stained cells were visualized by confocal laser scanning microscopy (CLSM) (Carl Zeiss, LSM700, Germany) using a 63 × objective lens. These experiments were also conducted in the presence of 5 mM Tu or 0.01% NaN_3_.

### Analysis of mitochondrial membrane potential (mtΔ*ψ*)

Rh123 is a fluorescence stain that distributes into the mitochondrial matrix directly in response to mtΔ*ψ*, without passage through the endocytotic vesicles and lysosomes [20]. We used Rh123 to investigate the effect of DD on the *C*. *albicans* mtΔ*ψ* alone or in the presence of 5 mM Tu or 0.01% NaN_3_. SC5314 cells were diluted to 1 × 10^6^ cells/mL in SD medium before exposing to different concentrations of DD and 8 mg/L of AMB (positive control) at 30°C. After the treatment for 3 h, the samples were stained with 5 μM Rh123 for 30 min incubation in dark [19]. The dark incubation for 30 min, the cells with different treatments were then washed and detected by flow cytometry. The obtained data were analyzed by WinMDI 2.9 software.

### Construction of green fluorescent protein tagged strain CAI4-*TOM70*-*GFP*

Auxotrophic mutant strain CAI4 was used to tag *TOM70* by homologous recombination of green fluorescent protein (GFP) sequences into the 3’ end of the *TOM70* open reading frame (ORF), as described previously [21, 22]. The cassette containing *GFP* and *URA3* selectable marker was amplified by primer (F: TAAAAATGTCCAATTTTAAATAATAAAATTGCTGAATTAATGAGACAAAGTGGTGCCATGGGTGGTAAAGGTGAAGAATTATT and R: AGAAAAAAAGTTAATAGGCAAGTAAGTAAGTAAGTAAGTAAGTAAGTAATTTAAATTTACTCTAGAAGGACCACCTTTGATTG). The PCR product was transformed into CAI4. After culturing on SD solid medium that did not contain uracil for 3 days, the colonies grown were identified by fluorescent microscopy (Olympus 1× 81 Olympus, Tokyo, Japan) and further confirmed by PCR. The positive colony was named as CAI4-*TOM70-GFP*.

### Effect of DD on the localization of Tom70-GFP

To uncover the effect of DD on the localization of Tom70-GFP, CAI4-*TOM70-GFP* was treated by various doses of DD with or without Tu and NaN_3_ at 30°C for 3 h in SD medium. The localization of Tom70-GFP was visualized using CLSM with a 63 × objective lens.

### Effect of sodium azide (NaN_3_) and thiourea (Tu) on antifungal activity of DD

SC5314 cells were adjusted to an inoculum concentration of 5 × 10^5^ cells/mL and pre-incubated with 5 mM of Tu or 0.01% of NaN_3_ for 1 h. DD with final concentration of 8 mg/L was added to the cultures with or without Tu or NaN_3_. DD free samples were regarded as control groups. The antifungal activity was determined as the present of viable cells comparing with drug free sample, by the colony counting method after the incubation for 3 h at 30°C. The results were represented as the mean values ± SDs and measurements were taken in triplicate of three independent assays. Statistical significances were determined by Student’s *t*-test and a *P* value < 0.05 indicated statistical significance.

## Results

### Effect of DD on the cell proliferation

The purity of DD used in this study was 98.5% as determined by high-performance liquid chromatography (Fig 1B). MTT assay showed that the IC_50_ values of DD against A549, A2780, MDA-MB-231, and HUVEC cell lines were 17.9, 19.3, 18.6, and 15.8 mg/L, respectively. Those of AMB were 21.5, 6.3, 19.3, and 10.3 mg/L, respectively (Table 2).

**Table 2:** The IC_50_ values of DD against human cell lines.

Cell lines	IC_50_ (mg/L)	
	DD	AMB
A549 (human lung adenocarcinoma cell line)	17.9	21.5
A2780 (human ovarian carcinoma cell line)	19.3	6.3
MDA-MB-231 (human breast cancer cell line)	18.6	19.3
HUVEC (human umbilical vein endothelial cell line)	15.8	10.3

### Effect of DD on the viability of *C*. *albicans* cells

DD displayed potent antifungal activity against five tested *Candida* species. The MIC_80_ values of DD against *C*. *albicans* (SC5314), *C*. *krusei* (CK3), *C*. *tropicalis* (CT2), *C*. *glabrata* (CG1), and *C*. *parapsilosis* (CP1) were 8, 32, 16, 32, and 16 mg/L, respectively, and those of AMB were 0.5, 1, 0.5, 0.5, and 0.5 mg/L, respectively (Table 3). The time-killing kinetics profile showed that DD (8 mg/L) reached its maximal fungicidal activity (84.0% ± 0.4%) within 30 min, more quickly than AMB (3 h) (Fig 1C). The data suggested that DD possessed a quick mode of fungicidal action against *C*. *albicans*.

**Table 3:** The MIC_80_ values of DD against *Candida* species.

Strains	MIC_80_ (mg/L)	
	DD	AMB
SC5314 (*C*. *albicans*)	8	0.5
CK3 (*C*. *krusei*)	32	1
CT2 (*C*. *tropicalis*)	16	0.5
CG1 (*C*. *glabrata*)	32	0.5
CP1 (*C*. *parapsilosis*)	16	0.5

### Effect of DD on cell membrane

The location and functional importance of cell membranes make them particularly susceptible to the toxic effects of chemicals. To uncover the effect of DD on the cell membrane, we detected the integrity of cell membrane, the change of intracellular osmotic pressure, the ultrastructure of cells, and the expression of genes involved in the biosynthesis of cell membrane accordingly.

#### Integrity of cell membrane

**Fig 2:**
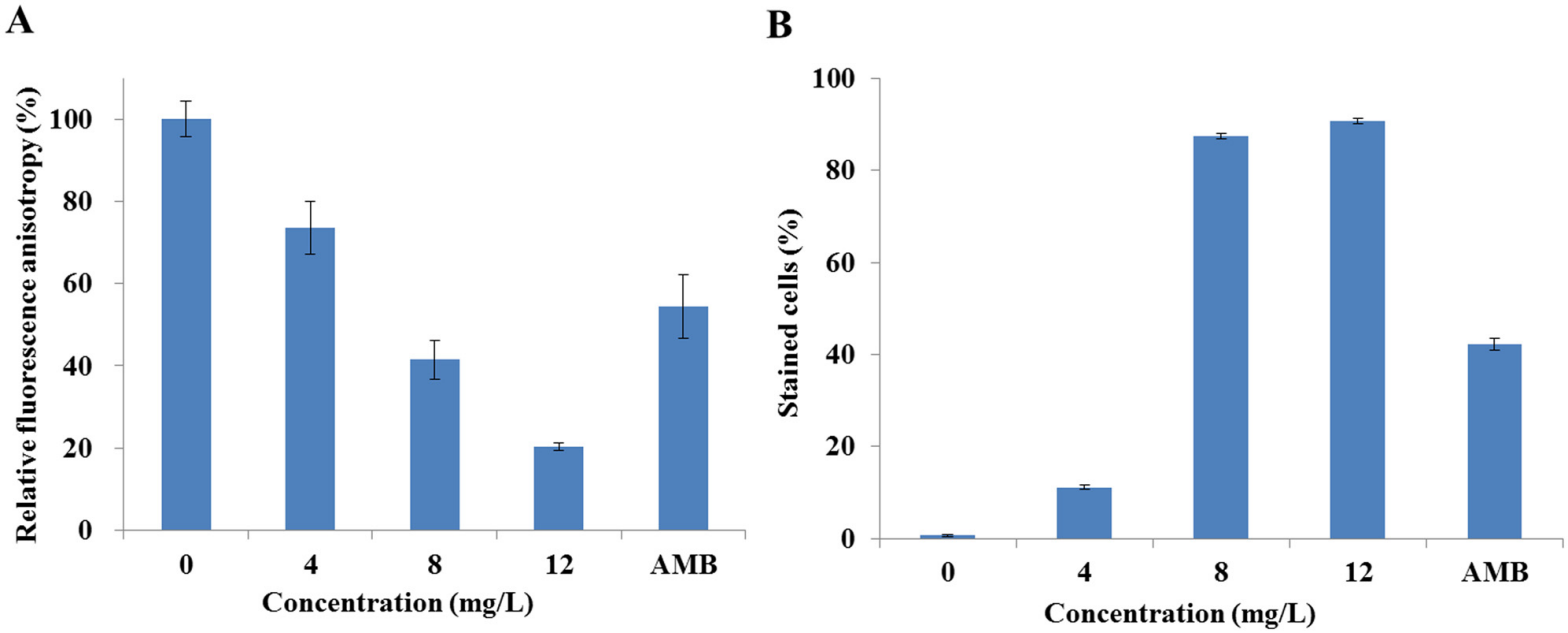
Effect of DD on the cell membrane integrity. (A) showed the effect of DD on the cell membrane dynamic. SC5314 cells were treated with various doses of DD or 8 mg/L of AMB (positive control) for 3 h following with staining of DPH for spectrofluorophotometer detection. (B) showed the change of membrane permeabilization after cells were treated with DD or 8 mg/L of AMB (positive control). Treated cells were stained with PI and analyzed by flow cytometry. The cells with more than 10^1^ fluorescence intensity were regarded as stained cells. Bars indicate standard deviations.

We firstly detected the effect of DD on the integrity of cell membrane by analyzing the dynamics and permeability of *C*. *albicans*’ cell membrane using fluorescent stains DPH and PI as indicators, respectively. The fluorescence intensity of DPH showed that DD abated the DPH fluorescence in a dose-dependent manner. The DPH fluorescence intensity was reduced to 73.5% ± 6.4%, 41.5% ± 4.7%, and 20.2% ± 1.0% compared with the vehicle control (100% ± 4.3%) when cells were treated with 4, 8, and 12 mg/L of DD, respectively. 8 mg/L of AMB treatment, the positive control, resulted in a reduction of 54.4% ± 7.8% (Fig 2A).

Analysis of the PI penetration using flow cytometry showed that DD treatment induced an increase of stained cells in a dose-dependent manner (Fig 2B). The drug free sample and positive sample had 0.7% ± 0.2% and 42.2% ± 1.3% of permeabilized cells (stained cells), respectively. Treatments with 4, 8, and 12 mg/L of DD resulted in 11.0% ± 0.5%, 78.9% ± 1.1% and 90.7% ± 0.5% of permeabilized cells, respectively.

DPH can easily associate with the hydrocarbon tail region of phospholipids in the cytoplasmic membrane without disrupting the intact of cell membrane. Decrease in fluorescence intensity indicates low structural order or high fluidity of cell membrane [23]. So, the decrease of DPH fluorescence intensity revealed the perturbation of the cell membrane by DD treatment. PI only penetrated damaged or permeabilized cell membranes, and stained cells emitted red fluorescence [15]. Therefore, the increased number of stained cells implied more cell membrane permeabilization induced by high doses of DD treatment. In this experiment, the changes of DPH fluorescence intensity and percentage of PI stained cells reflected that cell membrane was compromised by DD treatment.

#### Cell morphology and intracellular glycerol contents

**Fig 3:**
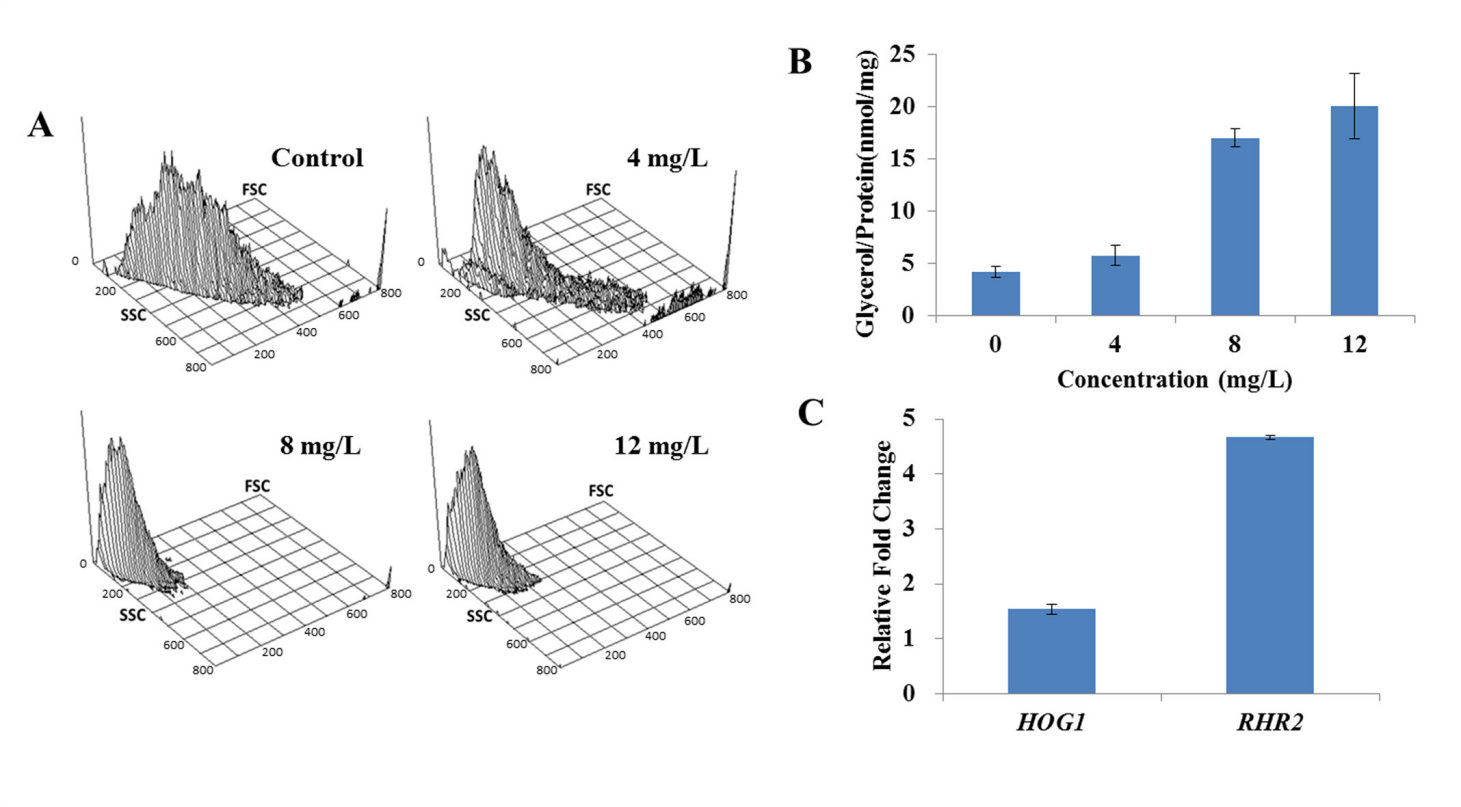
Effect of DD on the cell morphology and intracellular glycerol. (A) The alterations of cell morphology were analyzed by flow cytometry. FSC (y-axis) is an indicator of size, SSC (x-axis) is an indicator of granularity and the z-axis represents the cellular population intensity. The values of x-axis and y-axis are linear and provide relative values for comparison among different treatments. (B) The contents of intracellular glycerol were measured using Glycerol Assay Kits after treated with various doses of DD for 3 h. (C) The total RNAs were extracted by the hot phenol method and the expressions of *HOG1* and *RHR2* were measured using qPCR. Bars indicate standard deviations.

We then examined the morphology of cells by flow cytometry. The results, in which FSC (y-axis) was an indicator of size, SSC (x-axis) was an indicator of granularity and the z-axis represented the cellular population intensity, showed that homogeneous populations dominated the untreated sample. However, the addition of DD shifted the population to a low FSC and SSC area (Fig 3A), suggesting that the co-incubation of DD resulted in the cell shrinkage. The change of cell morphology might be attributed to the cell membrane damage. It is known that the high-osmotic stress can induce the accumulation of intracellular glycerol by activating the high-osmolarity glycerol (HOG) pathway, and the perturbation of cell membrane integrity may induce the change of intracellular osmotic pressure [24]. Therefore, the contents of intracellular glycerol were determined, and the results showed a remarkable increase from 4.2 ± 0.5 nmol/mg in vehicle treatment to 5.7 ± 1.0 nmol/mg, 17.0 ± 0.9 nmol/mg and 20.0 ± 3.1 nmol/mg under the treatments of DD (ranging from 4 mg/L to 12 mg/L) (Fig 3B). Moreover, the expression of HOG pathway genes, *HOG1* and *RHR2*, were determined by qPCR. *HOG1* plays a general role in regulating stress response in *C*. *albicans*, and *RHR2* is a glycerol 3-phosphatase gene involved in glycerol biosynthesis. When compared with the untreated control, the transcript levels of *HOG1* and *RHR2* in DD-treated group were upregulated by 1.54 ± 0.01 and 4.67 ± 0.04 folds, respectively (Fig 3C). These results suggested that DD treatment resulted in high-osmotic pressure stress.

The cell morphology change and intracellular glycerol accumulation further supported the conclusion that DD caused the membrane damage.

#### Transmission electron microscopy and gene expression

**Fig 4:**
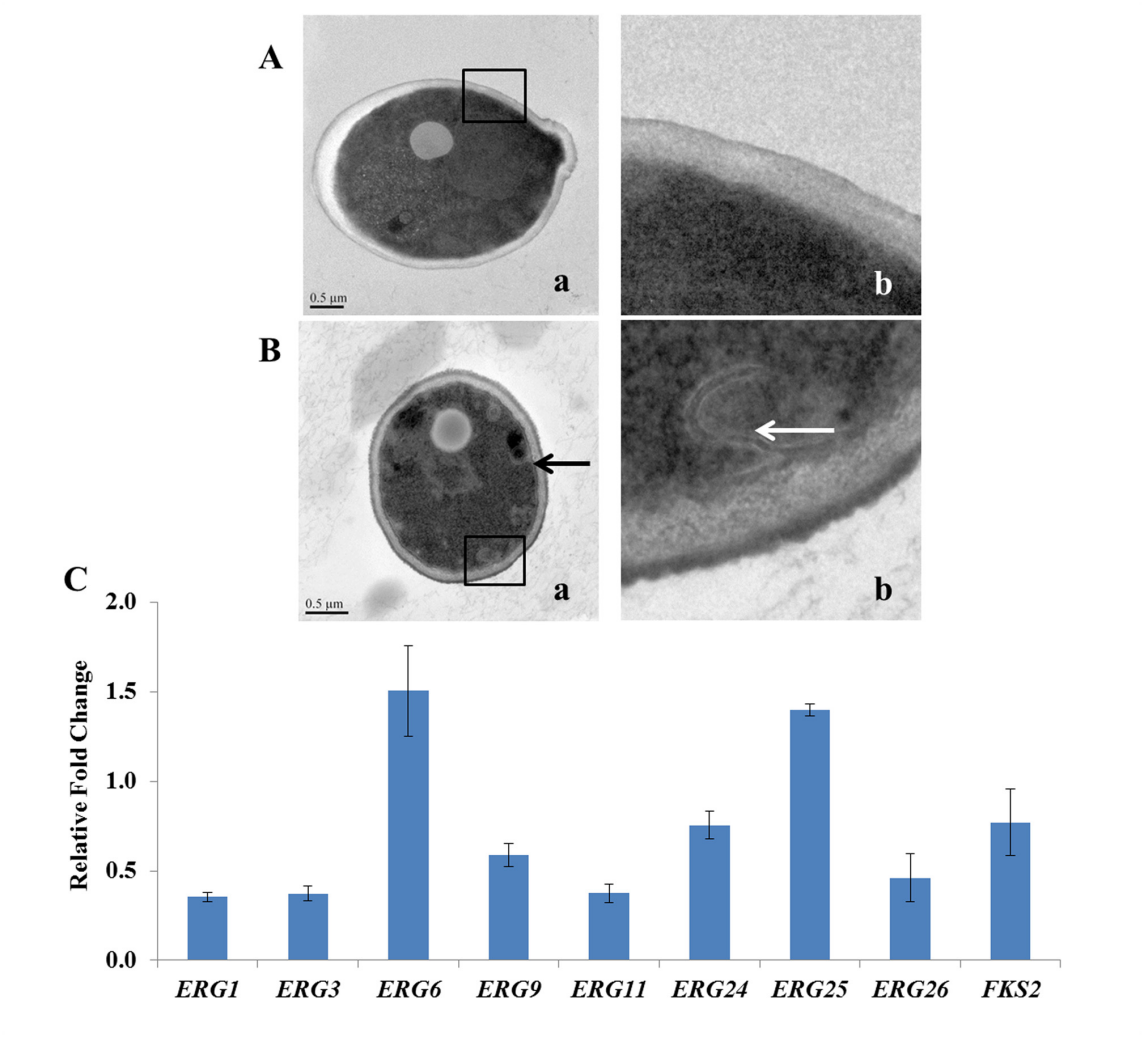
The ultrastructure of DD-treated *C*. *albicans* cells and the expressions of genes associated with cell membrane synthesis and cell wall assembly. (A) and (B) showed the transmission electron micrographs of *C*. *albicans* with 12 mg/L of DD-treatment or not. The destruction and fragmentation of plasma membrane were observed in DD-treated cells indicated by arrow in B. (b) is the magnification of (a). (C) showed the effect of DD on the expression of cell membrane related genes. After treatment with 8 mg/L of DD for 3 h, SC5314 cells were harvested for the total RNAs extraction and then the expressions of indicated genes were measured using qPCR. Bars in (C) indicate standard deviations.

TEM was utilized to reveal definitive ultrastructural features of the organisms in response to DD treatment. Untreated cells showed normal cellular morphology with a distinct cell membrane and intact membranous organelles. The cell membrane was smooth without membrane invaginations (Fig 4A). In contrast, the plasma membrane was completely destroyed in DD treated cells and no distinct cell organelles were observed (Fig 4B). It was notable that the cell membrane curled inward and formed a shape of ring or hook (shown by the arrow) under the DD stress (Fig 4B (b)).

We then quantified the expression of some genes (*ERG1*, *ERG3*, *ERG6*, *ERG9*, *ERG11*, *ERG24*, *ERG25* and *ERG26*) involved in the biosynthesis of ergosterol, which played an important role in the structure and function of cell membrane, and one gene *FKS2* in cell wall assembly. The results (Fig 4C) showed that most tested genes were down-regulated except *ERG6* and *ERG25*.

The ultrastructure provided direct evidence for the membrane damage and the down-regulation of genes expression supported this conclusion at the molecular level.

### Effect of DD on ROS production

ROS are the byproducts of cellular metabolism and primarily generated in the mitochondria. Cells produce more ROS under diverse and stressful conditions. If the production of ROS overwhelms the antioxidant capacity of cells, the extra ROS is likely to cause the cell damage.

#### ROS production

**Fig 5:**
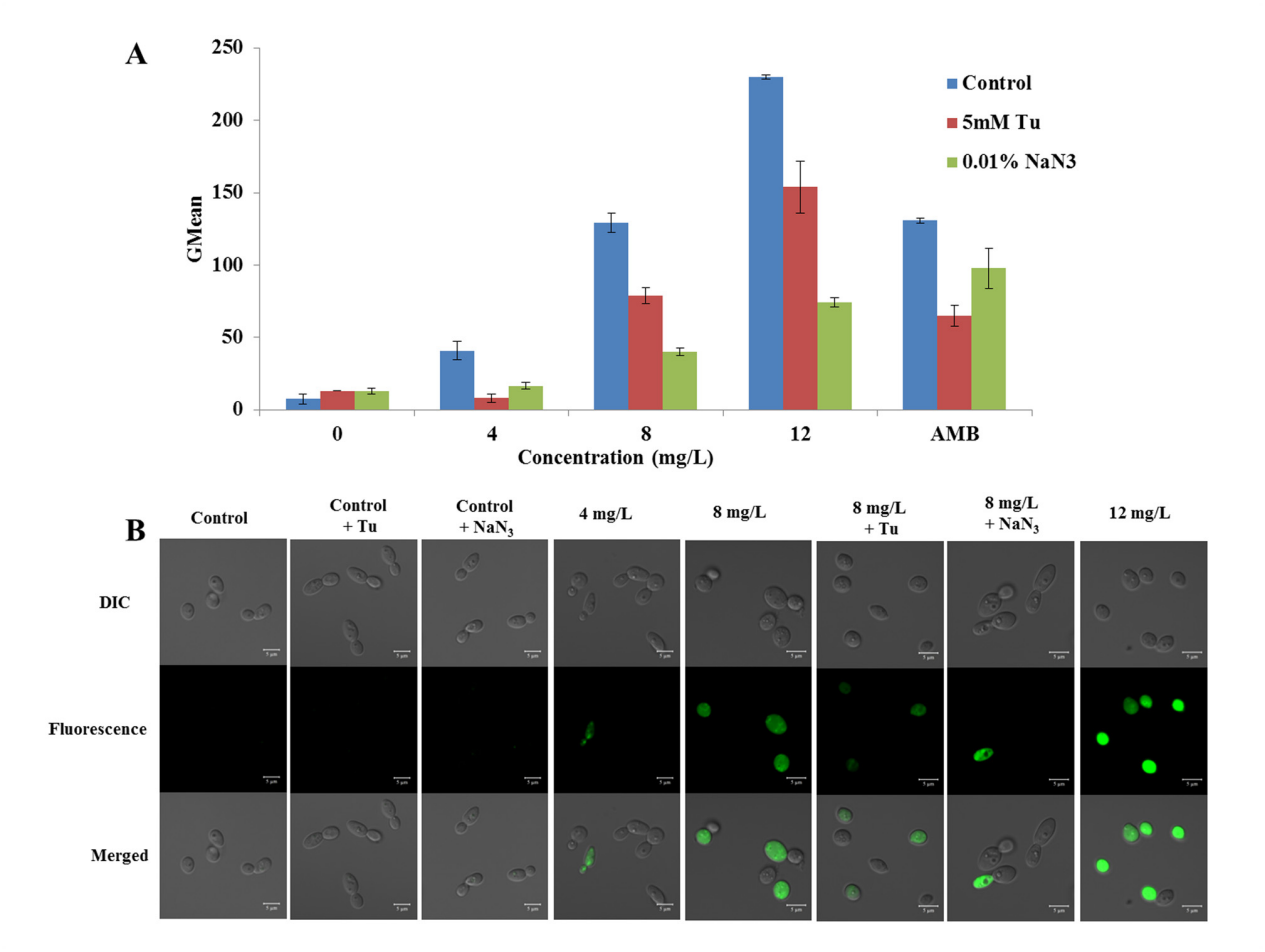
Effect of DD on intracellular ROS production. SC5314, preincubated with or without Tu or NaN_3_, was exposed to the increasing concentrations of DD or 8 mg/L of AMB (positive control) for 3 h. After staining with 40 mg/L of DCFH-DA, the samples were detected by flow cytometry and visualized by CLSM with 488 nm of excitation and 525 nm of emission. (A) showed the flow cytometry results and (B) showed the CLSM observation. The bars in (A) indicate standard deviations and in (B) indicate 5 μm.

The fluorescent probe DCFH-DA was utilized as a ROS indicator to evaluate the effect of DD on intracellular ROS production. The flow cytometry results showed that DD promoted ROS generation potently (Fig 5). The geometric mean (GMean) value was utilized to reflect the change of fluorescence intensity. Compared with the control group (GMean = 7.6 ± 3.5), 8 mg/L of AMB treatment increased ROS production by 17.2 ± 0.2 fold. DD stimulated ROS production by 5.4 ± 0.8, 17.0 ± 0.9, and 30.3 ± 0.2 folds at concentrations of 4, 8 and 12 mg/L, respectively, which is in accordance with the confocal microscopic observation (Fig 5C). The microscopic observation showed that treatments of DD at 8 and 12 mg/L resulted in more green fluorescence cells, indicating the elevated ROS production.

#### mtΔ*ψ* alteration

**Fig 6:**
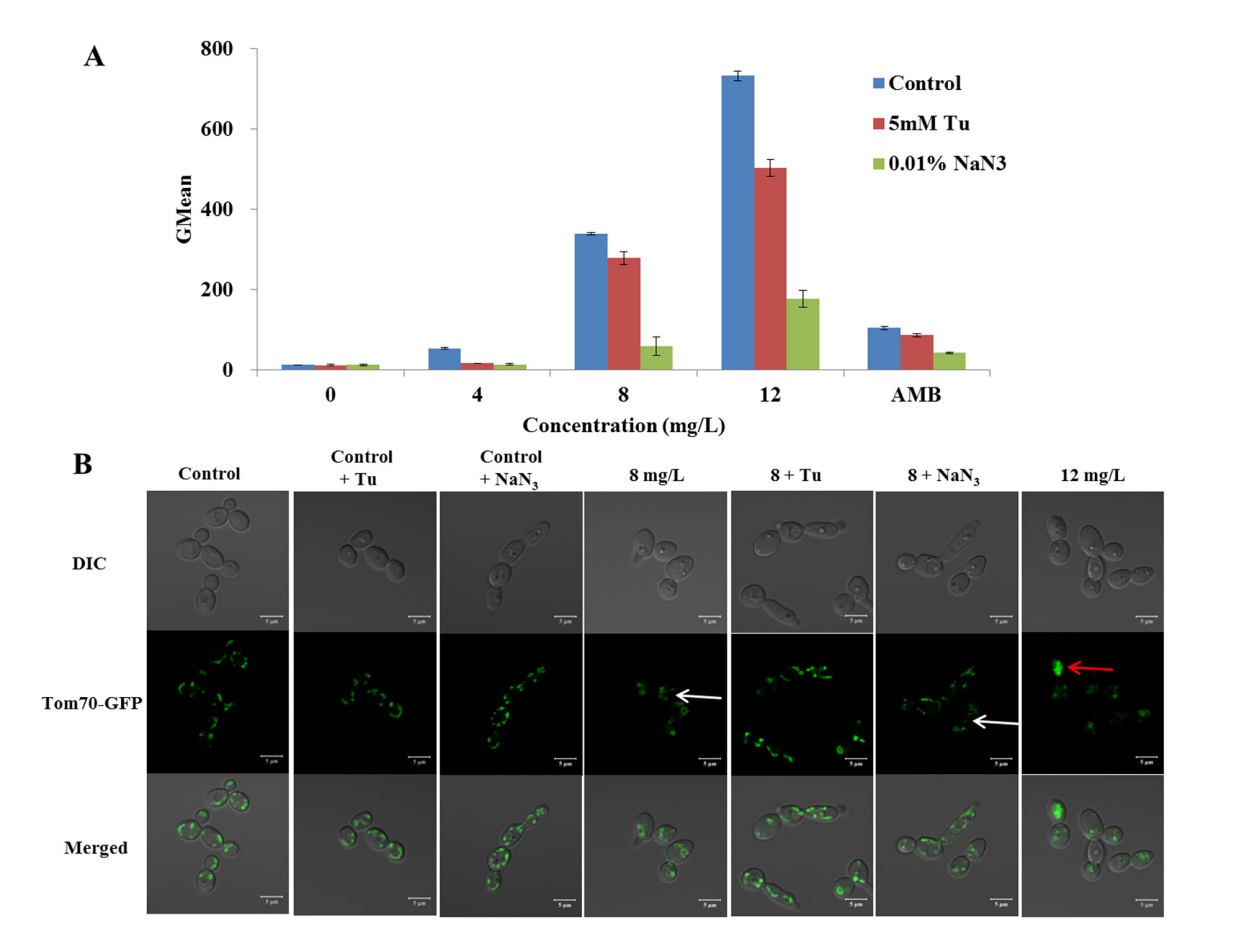
Effect of DD on the mitochondria function. SC5314, preincubated with or without Tu or NaN_3_, was treated with various concentrations of DD and 8 mg/L of AMB (positive control) for 3 h before staining with 5 μM of Rh 123 to indicate the alteration of mitochondrial membrane potential (mtΔ*ψ*). The fluorescence intensity was analyzed by flow cytomerty and shown at (A). *C*. *albicans* CAI4-*TOM70-GFP*, preincubated with or without Tu or NaN_3_, was treated with various concentrations of DD. The cells were visualized by CLSM and shown at (B). The bars in (A) indicate standard deviations and in (B) indicate 5 μm.

The mtΔ*ψ*, which is an indicator of the energetic state of the mitochondria, can be used to assess the activity of the mitochondrial proton pumps, electrogenic transport systems, and the activation of the mitochondrial permeability transition [25, 26]. Rh123, the potential-dependent distributional probe, was utilized to determine the mtΔ*ψ* of cells. In the study, we found that DD significantly increased the fraction of cells with high intensity of fluorescence (Fig 6A). DD increased the GMean value from 11.7 ± 0.3 (drug free control) to 53.3 ± 2.7, 338.7 ± 13.7, and 731.9 ± 12.6 at the concentrations of 4, 8, and 12 mg/L, respectively (Fig 6A). These results suggested that the treatment of DD increased the mtΔ*ψ* in *C*. *albicans*, indicating the dysfunction of mitochondria.

#### The location of mitochondria outer membrane protein Tom70

The TOM (translocase of the outer mitochondrial membrane) complex is responsible for the import of cytosolically synthesized mitochondrial preproteins into the organelle [27]. Tom70 is a protein of the TOM holo-complex and chiefly involved in the recognition of hydrophobic proteins of the inner membrane that carry internal targeting signals [28]. It displays a tubular structure when labelled with green fluorescent protein (GFP) under CLSM observation. However, when the dysfunction of the mitochondria occurs, Tom70 in *Saccharomyces cerevisiae* will become fragmented and ultimately aggregate or disperse [29, 30]. We constructed a GFP labelled strain CAI4-*TOM70-GFP* in *C*. *albican* to monitor the effect of DD on the mitochondria. CAI4-*TOM70-GFP* cells treated with 8 mg/L or 12 mg/L of DD presented the diffused (white arrow) or aggregated (red arrow) distribution of the Tom70-GFP protein (Fig 6B). The altered distribution of Tom70 confirmed the fact that DD induced the dysfunction of mitochondria.

#### The rescue effect of Tu and NaN_3_ on DD fungicidal activity

**Fig 7:**
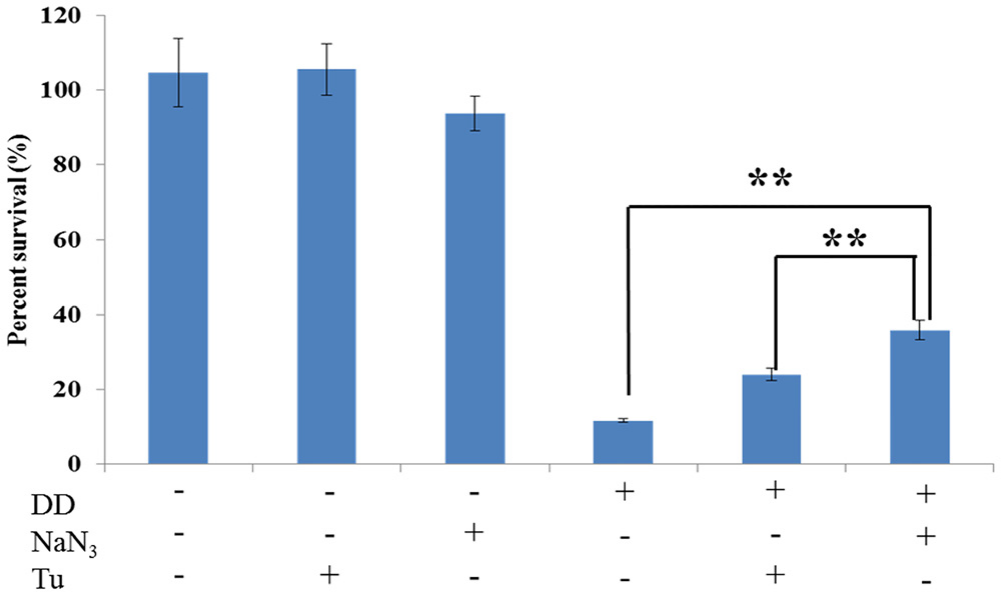
Effect of Tu and NaN_3_ on the fungicidal activity of DD. SC5314, pretreated with or without Tu or NaN_3_, was exposed to 8 mg/L of DD for 3 h at 30 °C. The number of viable cells was then determined by colony counting method. The bars indicate standard deviations. Statistical significances were determined by Student’s *t*-test. *P < 0.05. **P < 0.01.

To determine whether the ROS accumulation induced by DD is involved in the dysfunction of mitochondria and its fungicidal activity, we explored the scavenging effect of antioxidant, Tu, and mitochondria inhibitor, NaN_3_, on DD-induced ROS generation, mtΔ*ψ* alteration, mitochondria dysfunction, and fungicidal activity. As shown in Figs 5 and 6A, Tu and NaN_3_ have little effect on the ROS production and mtΔ*ψ* level in normal cell. However, the ROS generation and mtΔ*ψ* hyperpolarization were drastically retarded by the addition of Tu or NaN_3_ under DD treatment. In addition, the pre-incubation of Tu and NaN_3_ could protect the mitochondria from damage. Fig 6B showed that treatment with 8 mg/L of DD alone resulted in a diffused distribution of Tom70-GFP protein. By contrast, Tom70 in DD treated cells restored the normal localization in the presence of either Tu or NaN_3_. Moreover, the fungicidal effect of DD was partially counteracted by the addition of Tu or NaN_3_ (Fig 7). Tu or NaN_3_, which had little effect on the normal cells growth, significantly increased the survival percent more than 2 or 3 times (from 11.7% ± 0.5% to 24.0% ± 1.6% and 35.9% ± 2.7%) compared with the DD (8 mg/L) alone treated group, respectively. These results concluded that DD exerted its fungicidal activity, at least partially, through stimulating ROS formation.

## Discussion

The emergence of drug resistance highlights the importance of developing new therapeutics for fungal infections. Natural products either from microorganisms or plants are a rich source of new antifungal agents. Diorcinol D (DD), a diphenyl ether derivative extracted from endolichenic fungus, exerted antifungal activity against different *Candida* species.

We found that the MIC_80_ value of DD against *C*. *albicans* was lower than the IC_50_ values of DD for human cell lines. Of note is that it could reach its maximal activity within 30 min, much more quickly than that of AMB. These phenomenons promoted us to investigate its underlying molecular mechanisms.

The DPH and PI are the indicators of cell membrane kinetics and permeabilization. Present study showed that the treatment of DD decreased the fluorescence intensity of DPH and markedly increased the number of PI stained cells, which implied that the DD treatment damaged the integrity of cell membrane. Meanwhile, the shrinkage of DD treated cells, the observation of cell membrane damage, and down-regulation of the genes related to cell membrane biosynthesis, and cell wall assembly further provided evidences for the conclusion that DD caused the destruction of cell membrane.

The intracellular accumulation of glycerol, as an adaptive response, is essential for the survival of yeast under-high osmolarity conditions by activating the HOG pathway [31]. Since the accumulation of glycerol and the up-regulation of HOG pathway genes expression were prevalent in the treated cells, it was likely to conclude that DD induced cell membrane damage and further resulted in a high osmotic stress, which caused more glycerol production by activating the HOG pathway to release the stress. These results suggested that DD treatment disrupted cell membrane and induced high osmotic stress.

ROS, as a byproduct in cell metabolism, plays important physiological roles in the cell life cycle. However, if the ROS level exceeds its antioxidant capability, it may cause the metabolic disorder and eventually the cell death. A typical example is that fungicidal agent AMB stimulates ROS production and results in cell apoptosis or necrosis in *C*. *albicans* [32]. Mitochondria, as a major source of ROS production in the majority of eukaryotic cell types, are required for cellular energy production by oxidative phosphorylation, and the conserved processes such as iron metabolism [33], programmed cell death [34] and intermediary metabolism [35]. It is generally known that Tu, as an antioxidant, is able to neutralize intracellular ROS production, and NaN_3_, as an inhibitor of F_1_-ATPase involving in mitochondria function [36], can suppress the ability of mitochondria generating ROS. In this study, we found that both Tu and NaN_3_ could partially inhibit the resulted ROS accumulation, mitochondria dysfunction, and the fungicidal activity of DD. The protected effect of Tu and NaN_3_ on the *C*. *albicans* suggested that the decrease of ROS could relieve the stress on mitochondria, in other words, the dysfunction of mitochondria was attribute to the elevation of ROS accumulation. Taken together, it is concluded that DD stimulates mitochondria to produce extra ROS for anti-stress, which overwhelms the limit of normal cellular antioxidant capacity. The excess ROS damages the function of mitochondria in return and results in the alteration of mtΔ*ψ*, which ultimately contributes to the death of *C*. *albicans*.

Above all, our findings identified the potential mode of fungicidal action for DD. The destruction of cell membrane and the accumulation of ROS are two important factors responsible for the fungicidal activity of DD. DD probably initially damaged the cell membrane and resulted in high-osmotic pressure stress, which stimulated ROS production as a stress response. When the produced ROS exceeded cells’ antioxidant capability, the accumulated ROS further caused mitochondria dysfunction, cellular metabolic disorder, and cytoplasm membrane damage, which accelerated cell death. The two main factors contributing to the *C*. *albicans* cell death is probably applicable to the death of human cells [37,38]. This is likely to explain the similar values of DD against *C*. *albicans* and human cell lines.

Although the low therapeutic index of DD restricted it to be developed as a clinically antifungal drug, the quick fungicidal action still drew attention for further investigation. On one hand, therapeutic index of DD could be increased by the further chemical modification, just as the improved antineoplastic activity of riccardin D-N through aminomethylation of riccardin D in our previous research [37]. On the other hand, our published results showed DD could synergistically enhance the efficacy of FLC against *C*. *albicans* both in planktonic state as well as mature biofilms [39], which broadened the potential application of DD.

In sum, we elucidated the potential mechanisms of DD against *C*. *albicans*. It is the first time to elucidate the fungicidal mechanisms of a diphenyl ether derivative, which expands the current potential antifungal agents to combat fungal infections.
